# Diet-Dependent Modular Dynamic Interactions of the Equine Cecal Microbiota

**DOI:** 10.1264/jsme2.ME16061

**Published:** 2016-10-21

**Authors:** Camilla Kristoffersen, Rasmus B. Jensen, Ekaterina Avershina, Dag Austbø, Anne-Helene Tauson, Knut Rudi

**Affiliations:** 1 Department of Chemistry, Biotechnology and Food Science, Norwegian University of Life SciencesÅsNorway; 2 Department of Large Animal Sciences, Faculty of Health and Medical Sciences, University of CopenhagenFrederiksbergDenmark; 3 Department of Animal and Aquacultural Sciences, Norwegian University of Life SciencesÅsNorway

**Keywords:** cecum, carbohydrate, microbiota, short-chained fatty acids

## Abstract

Knowledge on dynamic interactions in microbiota is pivotal for understanding the role of bacteria in the gut. We herein present comprehensive dynamic models of the horse cecal microbiota, which include short-chained fatty acids, carbohydrate metabolic networks, and taxonomy. Dynamic models were derived from time-series data in a crossover experiment in which four cecum-cannulated horses were fed a starch-rich diet of hay supplemented with barley (starch intake 2 g kg^−1^ body weight per day) and a fiber-rich diet of only hay. Cecal contents were sampled via the cannula each h for 24 h for both diets. We observed marked differences in the microbial dynamic interaction patterns for *Fibrobacter succinogenes*, *Lachnospiraceae*, *Streptococcus*, *Treponema*, *Anaerostipes*, and *Anaerovibrio* between the two diet groups. Fluctuations and microbiota interactions were the most pronounced for the starch rich diet, with *Streptococcus* spp. and *Anaerovibrio* spp. showing the largest fluctuations. Shotgun metagenome sequencing revealed that diet differences may be explained by modular switches in metabolic cross-feeding between microbial consortia in which fermentation is linked to sugar alcohols and amino sugars for the starch-rich diet and monosaccharides for the fiber-rich diet. In conclusion, diet may not only affect the composition of the cecal microbiota, but also dynamic interactions and metabolic cross-feeding.

The horse has evolved a digestive tract that is capable of utilizing fibrous plant material as an energy source (14). The main functions of the hindgut (cecum and colon) are the microbial degradation and fermentation of fiber into energy-yielding products such as short-chained fatty acids (SCFA) (2), which have been suggested to provide approximately half of the energy demands of the horse (9).

Concentrates with starch-rich grain are traditionally fed to performance horses in order to meet the high energy demands of physical activity because starch-rich concentrates are more energy dense than fiber-rich forages. However, several studies have demonstrated that the feeding of starch-rich concentrates results in larger fluctuations in hindgut pH as well as SCFA concentrations and composition than fiber-rich forage (16, 23). The feeding of a high fiber diet was previously shown to result in greater microbial stability and reductions in lactic acid-producing bacteria and *Streptococcus* spp. in feces than in horses fed starch-rich concentrate (10, 41). A stable hindgut environment is crucial for the health of horses, and imbalances in the gut microbiota and fermentation products may lead to diseases such as colic (13) and laminitis (8, 27, 28). However, dynamic models need to be derived in order to understand the temporal stability of and fluctuations in the microbiota, as previously described for the human gut microbiota (38). We have used models for human infants in order to predict the colonization of the infant gut during the first 4 months of life (36). Dynamic models have also recently been used to explain ecological patterns in the murine gut (20). The most widespread and developed use of dynamic models is to describe interactions between larger animals such as the lynx and snow hare (34). To the best of our knowledge, dynamic interactions in the horse cecum microbiota in response to feeding have not yet been examined.

The aim of our study was to derive dynamic interaction models of the horse cecum microbiota for starch- and fiber-rich diets. We used cecum-cannulated horses to obtain samples from the same horses hourly for 24 h. We analyzed the microbiota taxonomically and functionally using Illumina sequencing. We also measured fluctuations in cecal pH and SCFA concentrations. In order to investigate dynamic properties, we used regression modeling in which the microbiota composition at a given time point was modeled based on that at the previous time point.

We observed marked differences in microbiota dynamic interactions between the diets evaluated.

## Materials and Methods

### Experimental design, animals, and feeding

**Fig. 1:**
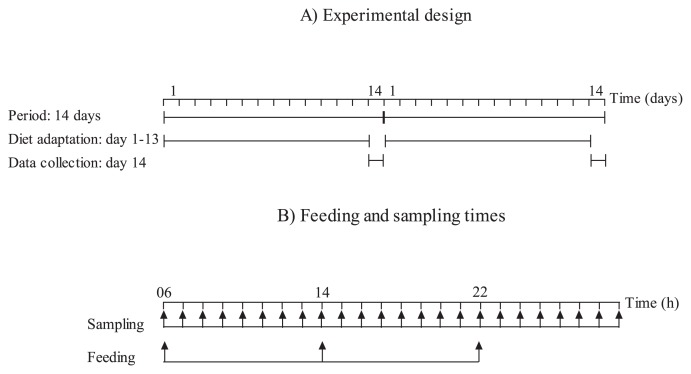
Experimental design (A) and feeding and sampling times during data collection (B) when feeding a hay only diet or hay supplemented with pelleted barley diet. Two horses were fed each diet in period 1 and then changed diets in period 2. The horses were fed three times every day, and 24 cecal samples were collected during data collection.

The 4 horses investigated remained healthy throughout the study, and were cared for according to the laws and regulations concerning experiments on live animals in Norway (*i.e.* the Animal Protection Act of December 20, 1974, and the Animal Protection Ordinance concerning Experiments on Animals of January 15, 1996). The experiment was performed as a crossover design with two experimental periods of 14 d in which the horses were fed two different diets (Fig. 1A). Each period ended with 24 h of measurements and sample collection such that the horses were fed the same diet 13 d prior to sample collection (Fig. 1B). Four cecum-cannulated Norwegian cold-blooded trotter horse geldings (age 7–17 years, bw 510–560 kg) were used in this study. They were housed in an unheated barn in 3×3 m individual stalls with wood shavings as bedding material. Throughout each experimental period, the horses were allowed access to a dirt paddock for approximately 6 h after morning feeding, except during measurements.

The horses were fit with a permanent polyvinyl chloride cannula (length ~15 cm; 40 mm o.d. and 30 mm i.d.) at the base of the cecum close to the ileo-cecal junction. Surgery was performed 2 to 12 years prior to experiments being conducted. The cannula was opened by removing a lid kept in place by a clamp. All experimental procedures were performed in compliance with the regulatory requirements that apply to the use of animals for scientific purposes in Norway, and were approved by the National Animal Research Authority.

The dietary treatments consisted of a hay only diet (HAY) and hay (74% of dry matter intake [DMI]) supplemented with a pelleted barley (26% of DMI) diet (BARLEY). DMI was 7.2 kg for both diets and nutrient intakes for the two diets are shown in Table 1. Two horses were fed the HAY diet and two were fed the BARLEY diet in the first period and they then changed diets in the second period. Hay was mainly mature timothy, and pelleted barley (Felleskjoebet Agri, Gardermoen, Norway) was produced by grinding barley to pass a 3-mm screen, mixing with molasses (4% of the pellet) and a binding agent (0.7% of the pellet; PellTech 2, Borregaard, Sarpsborg, Norway), and pelleting in an expander at 88°C. Three equal meals of hay were fed at 06:00, 14:00, and 22:00 when feeding the HAY diet. When feeding the BARLEY diet, pelleted barley was fed at 06:00 before hay to ensure that all pelleted barley was consumed first, and this was followed by three equal meals of hay at 06:15, 14:00, and 22:00. A vitamin and mineral blend (100 g d^−1^) (Champion Multitilskudd, Felleskjoebet Agri, Gardermoen, Norway) was included in the meal at 22:00 for both diets. Water was available *ad libitum* at all times.

### Sample collection

Samples of feedstuffs were collected on day 14 in each period and stored in sealed plastic bags for later analyses of chemical compositions. Twenty-four samples of cecal fluid (approximately 100 mL) were collected from each horse by connecting a 400-mL syringe to a 100-cm long tube (12 mm o.d. and 10 mm i.d.) fitted with ~50 holes (diam. 3 mm) at the last 39 cm of the tube. The tube was inserted through the cannula into the cecum reaching 90 cm into the horse. The tube was kept in place by a special airtight clamp, and closed with a small rubber plug (15). The first sample of cecal fluid was taken before feeding the morning meal at 06:00 and then at hourly intervals for 23 h after the morning meal on day 14 in each experimental period. The same sampling regime was followed for the two experimental periods.

Each of the 24 cecal samples from each individual horse was immediately analyzed for pH. In the analysis of SCFA, 9.5 mL of cecal fluid was mixed with 0.5 mL concentrated formic acid, while in the analysis of microbial composition and function, subsamples were mixed with S.T.A.R. buffer (Stool Transport and Recovery Buffer; Roche, Germany) in a 2:1 ratio in order to suppress bacterial growth and prevent the degradation of nucleic acids. Samples were then frozen at −40°C until further analyses.

### Feedstuff analyses

All feedstuffs from each period were milled through a 1-mm screen, and these samples were then used for each of the analyses described below. The DM content in feedstuffs was measured by drying to a constant weight (at 105°C for 24 h) and samples were incinerated at 550°C for 6 h in order to measure ash content. Nitrogen was measured using the Kjeldahl technique (Tecator-Kjeltec system 2400, Tecator AB, Höganäs, Sweden) and crude protein was calculated as N×6.25. The fat content of feedstuffs was assessed by petroleum ether extraction in an Accelerated Solvent Extraction system (ASE 200, Dionex, USA). Starch was analyzed using an enzymatic-colorimetric method according to modified AACC-method 76-11 (21). Neutral detergent fiber (NDF) was measured with an Ankom^220^ fiber analyzer (ANKOM Technology, Fairport, NY, USA) as described previously (25) using sodium sulfite, alpha-amylase, and ash correction (aNDFom), while acid detergent fiber (ADF) was assessed according to AOAC Method 973.18 with the modification that samples were not washed with acetone and were corrected for ash.

### Analysis of pH and short-chained fatty acids

Cecal fluid pH was immediately measured in each sample with a pH electrode (SenTix 41, WTW GmbH, Weilheim, Germany) and a subsample containing 0.5 mL concentrated formic acid/9.5 ml cecal fluid was stored at −20°C for the later analysis of SCFA composition. The pH electrode was calibrated (at pH 4 and 9) before and after the measurements.

The cecal samples from before feeding the morning meal and 2, 4, 5, 6, 8, 10, 12, 14, 16, 18, 20, and 22 h after were analyzed for SCFA using a MaxMat PL II Multi-purpose diagnostic gas chromatography (GC) analyzer system (MaxMat S.A., Montpellier Cedex, France) at the NMBU center for animal experiments (Ås, Norway), which routinely performs this analysis. The results obtained were reported as total SCFA concentrations (mmol L^−1^) with acetate, propionate, and butyrate being expressed as proportions of total SCFA (mol 100 mol^−1^).

### DNA isolation

Genomic DNA was isolated from samples conserved in STAR buffer using a MagLGC^TM^ Total Nucleic Isolation Kit (LGC, London, UK). In order to ensure the disruption of cell walls, samples were first subjected to mechanical lysis using glass beads. Samples were thawed and homogenized by vortexing, and 300 μL of the sample was then transferred into a micro tube (Sarstedt, Nümbrecht, Germany) with acid-washed glass beads (<106 μm, 0.1 g) (Sigma-Aldrich, St. Louis, Missouri, USA). All the tubes were processed twice in the MagNa Lyser (Roche, Basel, Switzerland) at 2,000 rpm for 40 s with a 40-s rest between runs. Samples were kept cold during rest to prevent DNA degradation. Tubes were then centrifuged at 13,500 rpm for 5 min.

In order to remove cellular proteins, 50 μL of lysis buffer and 5 μL proteinase (25 mg mL^−1^) were added to 50 μL of the supernatant. The samples were then incubated in the KingFisher Flex robot (ThermoScientific, Waltham, MA, USA) at 55°C for 10 min.

All steps were performed according to the manufacturer’s recommendations. DNA extraction was controlled by qPCR targeting the 16S rRNA gene using universal prokaryotic primers (27) and by Qubit measurements (Life Technologies, Carlsbad, CA, USA). In each of the 5 DNA extraction runs (due to the size of the dataset, extractions were performed in 5 batches), we included two extraction controls consisting of a mixture of 5 random samples. The same controls were used in all DNA extraction runs. These controls were used to capture the technical variance between the runs introduced throughout the whole analytical process.

### Illumina sequencing

Genomic DNA was amplified with the 16S rRNA gene primer pair PRK covering the variable regions V3 and V4 (42) using a two-step PCR approach in order to increase the amplification success rate. In the second PCR run, primers were modified by adding Illumina-specific adapters (29). The library consisted of 16 differently indexed forward primers and 36 differently indexed reverse primers, which made a total of 16×36=576 possible primer combinations for unique sample indexing.

Each PCR reaction contained 1.25 U HotFirePol DNA polymerase, 1×HotFirePol buffer B2, 2.5 mM MgCl2, 200 μM dNTPs (Solis BioDyne, Tartu, Estonia), 0.2 μM PRK341F and PRK806R primers (Life Technologies), and 1 μL of template DNA (ranging from 1 to 10 ng). Amplification was performed using a 2720 Thermal Cycler (Life Technologies) with initial denaturation at 95°C for 15 min and 25 cycles of denaturation at 95°C for 30 s, annealing at 50°C for 30 s, and elongation at 72°C for 45 s. Polymerization was completed at 72°C for 7 min.

The PCR products were then diluted 1:100, and these dilutions were used in the second PCR amplification step with a unique PRKillumina primer combination for each sample. In this step, 10 cycles were used and the annealing time was increased to 1 min in order to ensure the annealing of long primer oligonucleotides.

Gel electrophoresis results were used to normalize the PCR product library due to the presence of primer dimers. All samples were ranged by gel band strengths (strong, medium, and weak) and pooled (2 μL, 5 μL, and 10 μL for strong, medium, and weak bands, respectively). An E.Z.N.A Cycle-Pure kit (Omega bio-tek, Norcross, GA, USA) was used to purify the mixed PCR products twice. Samples were then sent to the Norwegian Sequencing Center for sequencing on a MiSeq platform (Illumina, San Diego, CA, USA) using 300-bp paired-end sequencing with the v3 kit.

In order to investigate the gene content in the samples, a shotgun metagenomic analysis with a Nextera XT DNA sample preparation kit (Illumina) was performed.

The Nextera XT DNA sample preparation kit (Illumina) was used for shotgun metagenome sequencing according to manufacturer’s recommendations with some exceptions. As recommended, the DNA library was purified with AMPure XP beads (Beckman Coulter, Indianapolis, IN, USA) to remove the remaining nucleotides and primer dimers as well as to select PCR fragments of the desired length. However, the amount of AMPure XP beads (Beckman Coulter) was increased to a ratio of 1:1. Furthermore, samples were normalized based on DNA concentrations and measured by Qubit instead of bead-based normalization recommended in the manual. Ten nanograms of DNA from each sample was added to the library pool and then sequenced (paired-end 300 bp with the v3 kit) on a MiSeq sequencing platform (Illumina).

### Data analysis

Time-series data were analyzed as repeated measurements using the MIXED procedure in SAS (Version 9.3, SAS, Cary, North Carolina, USA), in which the model comprised the fixed effects of diet, time, and their interactions, and the interaction of horse and period was included as a random effect. Serial correlations over horses were modeled using a spatial Gaussian correlation structure. Results are presented as least square (LS) means, and the standard error of the mean (SEM) is reported. Pairwise comparisons were made using the PDIFF option, and effects were considered significant if *p*<0.05. We evaluated whether distributions were normally distributed using the Anderson-Darling test for normality, while power calculations were performed taking into account differences in means, variance, and sample size (MINITAB, State College, PA, USA).

16S rRNA gene forward reads (300 bp) were analyzed using QIIME version 1.7.0 pipeline (5). Sequences remaining after quality filtering were clustered using the closed-reference *uclust* search (6) at the 99% identity level against the Greengenes v.13.5 database (22). The rationale for using a closed reference search is to avoid potential artificial sequences and to have well-defined Operational Taxonomic Units (OTUs). Due to a higher error rate, reverse reads were used to verify the phylogeny derived from the forward read. This was performed by the independent determination of the sequence phylogenies of both the forward and reverse reads using a PCA-based approach recently developed by us (3), with subsequent correlation analyses for the first principal component (PC). A strong correlation indicates that the forward and reverse reads capture the same phylogenetic information. We used total observed OTUs for alpha-diversity measurements and Weighted Unifrac for beta-diversity, while microbiota coverage was calculated using Good’s coverage (19). A Principal Component Analysis (PCA) was used to search for OTUs that explain most of the variance in the microbial consortia. PCA was performed using PLS toolbox (Eigenvector) running in the Matlab R2014b programing environment (Mathworks.). Of these, dominant OTUs (>1% of the total bacterial load) were selected to search for temporal trends. The OTU table and mapping files are available at www.nmbu.no/en/about-nmbu/faculties/vetbio/departments/ikbm/research/midivlab/archive/.

The unassembled shotgun metagenomic sequencing results were uploaded to and analyzed in MG-RAST (26) for organismal and functional classification. Default settings were used with a maximum e-value of 1e–5, minimum identity of 60%, and minimum alignment length of 15 amino acids. The Subsystems database was used for functional annotation (30), and the KEGG mapper for identifying metabolic pathways (17). Data have been made publicly accessible through the link metagenomics.anl.gov/linkin.cgi?project=7746.

Dynamic models were derived by linear regression models using data at time t as responses, and data at time t-1 as predictors (TIBCO S+, TIBCO, USA). Only significant predictors (*p*<0.05) were considered in the models. Associations between functional subsystems and taxonomic groups were assessed by Partial Least Square (PLS) regression analyses (Eigenvector), while correlations were evaluated by Pearson’s correlation coefficient. Correlations (*p*<0.05) with coefficients >0.5 were used for network analyses (Matlab, Mathworks, USA).

## Results

### Feedstuffs and feed intake

The DM contents in hay and barley were 88.9% and 87.0%, respectively, while nutrient contents were (in % of DM) ash: 4.9 and 2.1%, fat: 2.1 and 1.2%, CP: 8.3 and 8.4%, starch: 0 and 49.9%, NDF: 55.3 and 14.2%, and ADF: 30.7 and 3.6%, respectively. The DM and nutrient contents of the feedstuffs were used to calculate DM and nutrient intakes in each meal and each d as shown in Table 1. DM, ash, fat, and CP intakes were similar for the two diets, whereas starch intake was higher for horses fed the BARLEY diet than for those fed the HAY diet. NDF and ADF intakes were higher for horses fed the HAY diet than for those fed the BARLEY diet. Horses consumed all the feed offered, and there were no feed refusals.

### SCFA and pH

**Fig. 2:**
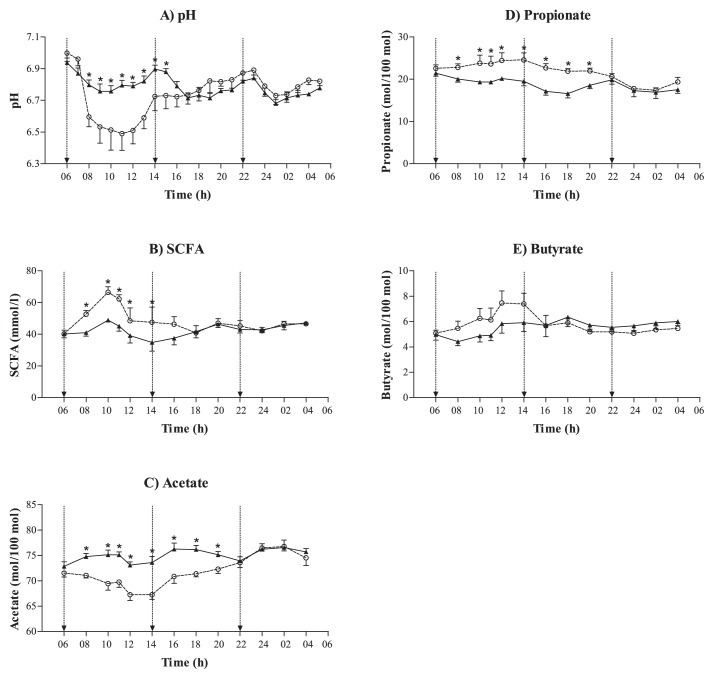
Time-series measurements of pH (A), total SCFA (mmol l^−1^) (B), and molar proportions (mol 100 mol^−1^) of acetate (C), propionate (D), and butyrate (E) when feeding a hay only diet (solid line) or hay supplemented with pelleted barley diet (dotted line). Time refers to the time of day and vertical lines represent feeding times. Asterisks associated with the graphs indicate a significant diet difference at the specific time point.

The effects of diet and time on cecal pH and SCFA data are presented in Fig. 2. Cecal pH decreased after feeding, and an interaction of diet and time (*p*<0.001) was present when pH was decreased more by the feeding of barley than hay. A rapid decrease in cecal pH was measured 2 h after feeding the BARLEY diet. The total SCFA concentration in the cecum increased over time reciprocal to pH, and the BARLEY diet reached higher concentrations than the HAY diet after feeding barley in the morning. In both diets, the molar proportion of acetate in the cecum was the greatest, followed by propionate and butyrate. The molar proportion of acetate was lower, whereas that of propionate was higher after feeding barley in the morning than hay. The effect of the 06:00 meal of barley persisted until 22:00 in the evening, *i.e.* for 16 h. The molar proportion of butyrate was more stable when feeding the HAY diet than the BARLEY diet, and a slightly positive increase was observed with time.

### Microbiota composition

Comparisons of the forward and reverse reads for phylogenetic information showed a Pearson’s correlation of 0.84 for the first PC, suggesting a similar phylogenetic content. Due to the lower error rate (median Q score=30 for positions 250 to 300) for the forward read than for the reverse read (median Q score=24 for positions 250 to 300), we only continued with the forward read in subsequent analyses. 16S rRNA gene sequencing for the forward read generated an average of 10056±7994 (average±standard deviation) for each sample after quality filtering and chimera removal. More than 90% of the samples contained more than 2,000 reads; therefore, this was set as the limit for further analyses. We detected a total of 7,769 OTUs in these samples, with a squared regression coefficient of 0.89±0.02 (average±standard deviation) for the control samples used to cover technical variations. The average number of OTUs observed in each sample was 473±62 (average±standard deviation). The overall Good’s coverage was 84.6±3.0 (average±standard deviation) for the dataset. Rarefaction analyses support relatively good coverage (Suppl. Fig. S1). Regarding diet, approximately 80% of the OTU distributions were explained by a normal distribution (Anderson-Darling test). The Weighted Unifrac beta-diversity between horses (0.20±0.07) was only slightly higher than that within each horse (0.15±0.02), while no significant differences were observed between the two feeding periods. The overall dominating phyla were *Firmicutes* and *Bacteroidetes*. The phyla *Tenericutes*, *Spirochaetes*, *Cyanobacteria*, and *Fibrobacters* showed significant mean differences between diet groups (Suppl. Fig. S2). No diet effects were found for observed species, while Weighted Unifrac analyses showed significantly (*p*<0.001) lower beta-diversity (0.18±0.06) for horses fed the BARLEY diet than for those fed the HAY diet (0.21±0.08). The PCA analysis revealed that 11 OTUs within the phyla *Firmicutes*, *Fibrobacters*, and *Spirochaetes* were the most influential with respect to temporal development and/or dietary differences as assessed by their large loadings (Suppl. Fig. S3; Suppl. Table S1).

**Fig. 3:**
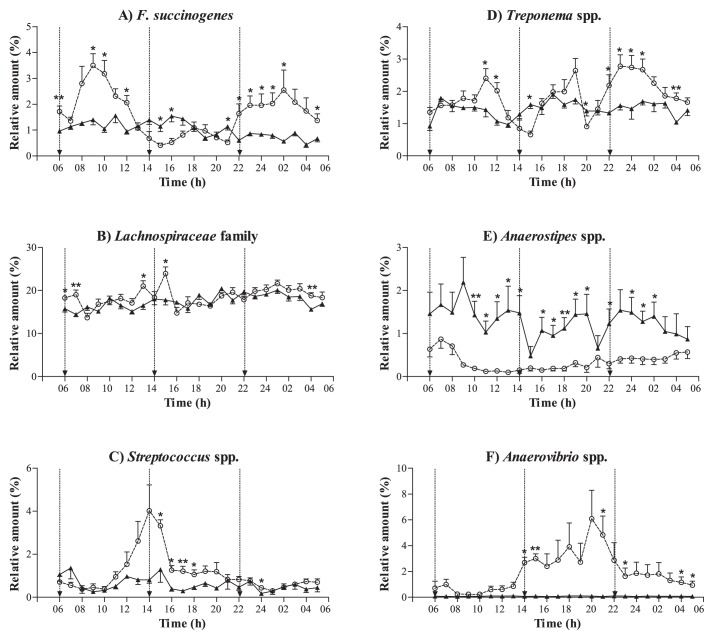
Times-series measurements of relative amounts of main phylogroups when feeding a hay only diet (solid line) or hay supplemented with pelleted barley diet (dotted line). Time refers to the time of day and vertical lines represent feeding times. Asterisks associated with the graphs indicate a significant diet difference at the specific time point.

Although the *Lachnospiraceae* family (represented by 4 OTUs) remained relatively stable in each diet group (Fig. 3B), its relative abundance differed significantly between the tested diets (*p*=0.004). *Fibrobacter succinogenes* (represented by 1 OTU) showed a major peak in its abundance at 4 h post feeding for the BARLEY diet (Fig. 3A), while *Streptococcus* (represented by 2 OTUs) had the highest abundance at 8 h post feeding (Fig. 3C). *Treponema* (represented by 3 OTUs) showed a significant diet difference (*p*<0.001), with temporal fluctuations for horses fed the BARLEY diet (Fig. 3D). *Anaerovibrio* (represented by 1 OTU) showed a significant diet difference (*p*<0.001), with a broad peak between 7 and 17 h post feeding (Fig. 3F). Significantly higher amounts of *Anaerostipes* (represented by 1 OTU) were detected with the HAY diet than with the BARLEY diet (*p*<0.001), with relatively constant amounts being detected during the course of the experiment (Fig. 3E).

### Dynamic interactions of microbiota, pH, and SCFA

**Fig. 4:**
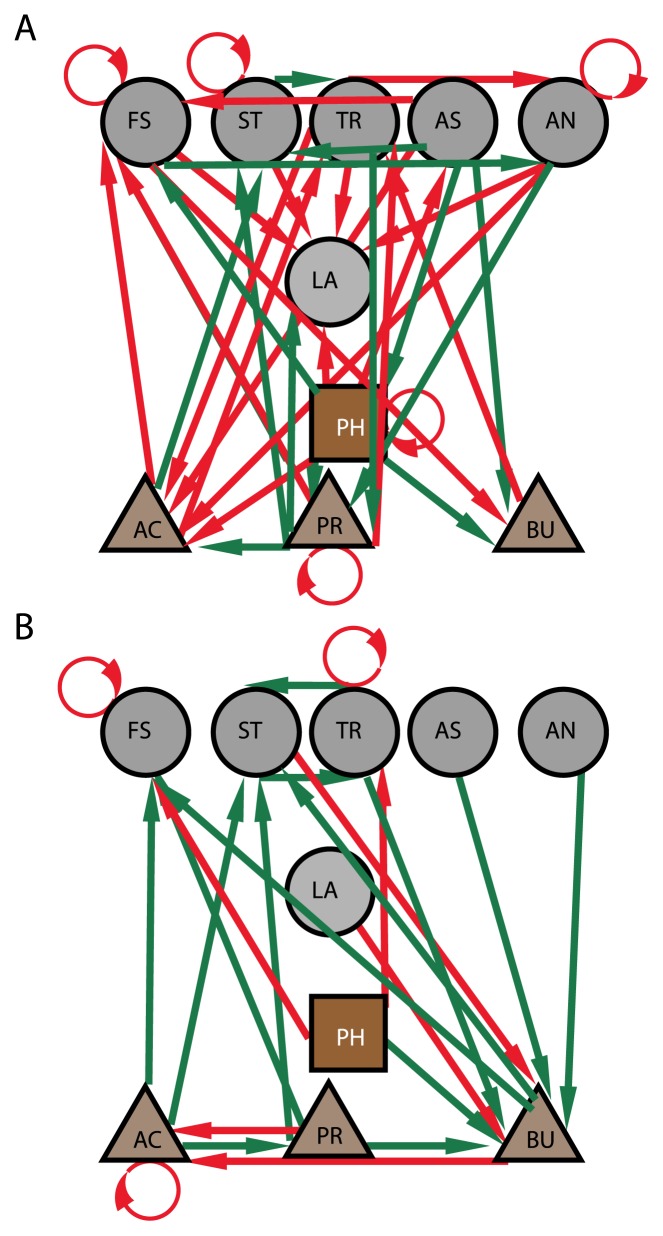
Dynamic interactions in the horse cecum for BARLEY (A) and HAY (B) diets. Dynamic interactions are represented with arrows, with direction representing the direction (the category at the start affects the category at the arrowhead) and color representing whether the interaction is positive (red) or negative (green). Only significant interactions (*p*<0.05) for regression coefficients >|0.3| are shown. Numeric values for interactions are shown in Suppl. Fig. S4. The following abbreviations are used: LA: *Lachnospiraceae*, AS: *Anaerostipes*, AN: *Anaerovibrio*. ST: *Streptococcus*, FS: *Fibrobacter succinogenes*, TR: *Treponema*, AC: acetate, PR: propionate, BU: butyrate, and PH: pH.

Marked differences were observed in the dynamic interactions of microbiota composition and SCFA for the two diet groups (Fig. 4; Suppl. Fig. S4). Microbial interactions were slightly stronger for horses fed the BARLEY diet than for those fed the HAY diet. Among horses fed the BARLEY diet, the *Lachnospiraceae* family appeared to be central for bacterial interactions (Fig. 4A). Furthermore, an increase in acetate appeared to be strongly stimulated by *Lachnospiraceae*, while changes in propionate and butyrate were negatively influenced by the bacterial components *Treponema* and *Anaerostipes*, respectively (Fig. 4A). In the HAY diet, the *Lachnospiraceae* family appeared to have switched to a positive interaction with butyrate, while the acetate interaction was neutral. These bacterial components only explained a minor portion of the changes observed in biotic and abiotic factors (Fig. 4B).

### Functional networks

A total of 8,507,537 sequences with a mean sequence count of approximately 350,000 sequence reads in each sample (*n*=24) and an average read length of 188 base pairs was obtained from shotgun metagenome sequencing. Approximately 5% of the sequence reads failed to pass the quality control of MG-RAST. Among quality control-passed sequences, 0.2–0.8% contained rRNA genes, approximately 38% contained predicted proteins with known functions, and approximately 47% contained predicted proteins with unknown functions. Furthermore, approximately 9% of the quality control-passed sequence reads had no rRNA gene or predicted proteins. Bacteria accounted for most of the reads with 88.82%±0.13 (average±standard deviation), while Eukaryota only represented 0.68%±0.03, Archaea 0.46%±0.0, and Viruses (0.08%±0.01). Similar bacterial phyla were examined by 16S rRNA gene and shotgun sequencing, while the composition obtained showed marked differences. *Fibrobacteres* was the only phylum that showed the same distribution by both approaches (Suppl. Fig. S5).

Twenty-eight subsystems were detected by MG-RAST, for which clustering-based subsystems of unknown functions were the most abundant and carbohydrate metabolism was the second most abundant. A comparison of the two diets for the time point with the lowest pH by the KEGG map showed that dietary differences were associated with carbohydrate and lipid metabolism (Suppl. Fig. S6). Pathways for inositol metabolism were associated with the BARLEY diet, while the HAY diet was associated with fatty acid chain elongation.

**Fig. 5:**
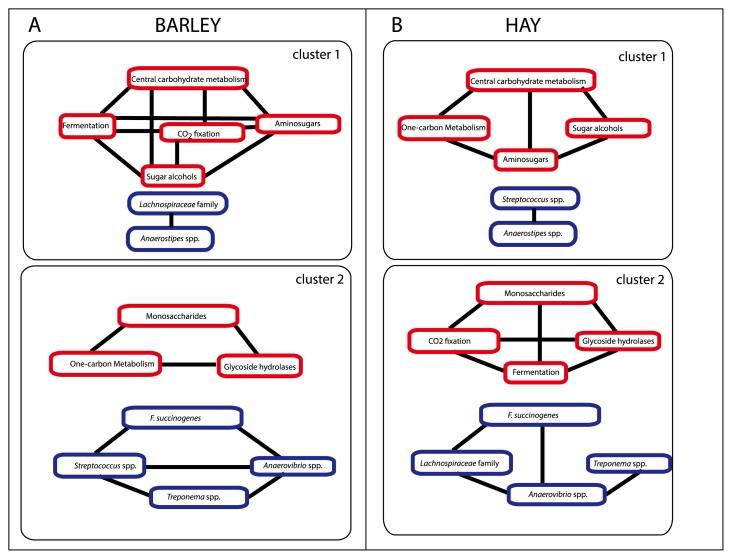
Interaction patterns in carbohydrate metabolism when feeding hay supplemented with barley (A) or hay only (B) diets. Red boxes indicate functional subsystems, while blue boxes indicate main taxonomic groups. Black lines between the boxes indicate Pearson’s correlation >0.5. The boxes marked clusters represent co-occurring metabolic and taxonomic clusters, as assessed by a multivariate regression.

Eight out of the 12 carbohydrate metabolism subgroups showed significant differences either between the two diets or between the time points sampled (Suppl. Table S2 and S3). The PLS multivariate regression showed that the differences detected in the 8 subgroups may be predicted based on the 6 bacterial groups showing the largest temporal fluctuations (Suppl. Fig. S7A and S8A). Based on regression loadings (Suppl. Fig. S7B and S8B), we found that the BARLEY diet and HAY diet showed two clusters of correlating subsystems (Fig. 5). However, there was a switch in correlation patterns; fermentation and CO_2_ fixation correlated with central carbohydrate metabolism for the BARLEY diet, whereas the correlation was switched to monosaccharides and glycoside hydrolases for the HAY diet.

The *Lachnospiraceae* family was consistently associated with fermentation and CO_2_ fixation for both diets, while *Streptococcus* was consistently associated with one-carbon metabolism and *Anaerostipes* was associated with central carbohydrate metabolism, amino sugars, and sugar alcohols. On the other hand, *F. succinogens*, *Treponema*, and *Anaerovibrio* were associated with monosaccharides and glycoside hydrolases (Fig. 5).

## Discussion

A systematic response to feeding was observed in the composition and function of the microbiota in the four horses investigated in the present study. Furthermore, beta-diversity between the horses was low, indicating a related microbiota. This is most likely due to long-term housing in the same environment (12). Therefore, it is likely that there are generally valid interaction patterns across horses housed in the same environment. Similar systematic interaction patterns have previously been observed in fermenters with a defined microbiota of a gut origin (37). These findings support the importance of bacteria-bacteria interactions in explaining the gut microbiota ecology.

The amount of substrates for fermentation was similar for the two diets, whereas the fluctuations observed in fermentation end products, pH, and the relative amounts of the main phylogroups of microbiota were larger in horses fed the BARLEY diet than in those fed the HAY diet. This was also reflected in the beta-diversity analyses. The small intestinal digestibility of barley starch was previously reported to be 75% (4). Given this digestibility, 260 g starch reached the hindgut for fermentation from the morning meal for the BARLEY diet. Starch may have caused larger fluctuations because it is more readily fermented than NDF from fiber (1).

*Streptococcus* and *Anaerovibrio* showed the largest fluctuations in horses fed the BARLEY diet. Using the dynamic model, we found that *Streptococcus* promoted its own increase while negatively interacting with the *Lachnospiraceae* family. On the other hand, the *Lachnospiraceae* family showed positive interactions with all bacterial groups, except itself and *Anaerovibrio*. Since *Streptococcus* promotes its own growth, preventing the outgrowth of this bacterial group is likely to be crucial for microbiota stability. *Streptococci* are strongly associated with lactate build-up and an acidotic state (27, 32). *Lachnospiraceae-*related clostridia have been shown to ferment lactate to propionate and acetate (39). Therefore, the *Lachnospiraceae* family appears to play a major role here. This family has also been identified as the most widespread and stable component of the human gut microbiota (33). Although *Anaerovibrio* showed a major temporal fluctuation, this group did not appear to affect any of the other bacterial groups, and may not be important for the overall stability of the microbiota.

Marked differences were observed in bacterial interaction patterns between the diets, with the most pronounced difference being that the *Lachnospiraceae* family had a central role in the interactions observed in horses fed the BARLEY diet, but not the HAY diet. This may be explained by its association with fermentation and CO_2_ fixation genes, which is consistent with the observation that this family is one of the main SCFA producers in the gut (11, 24). Moreover, species belonging to this family are known to harvest energy by the assimilation of CO_2_ and hydrogen (31), which are major end products of rapid fermentation, as observed for horses fed the BARLEY diet. The lower fermentation rate in horses fed the HAY diet may explain why *Lachnospiraceae* is not central here. The slower fermentation for HAY is most likely due to sugars being derived from cellulose or other polysaccharides with slow degradation rates. This is consistent with the metabolic connection of *Lachnospiraceae* and *F. succinogenes* for the HAY diet. *F. succinogenes* is a cellulolytic bacterium associated with monosaccharide and glycoside hydrolases in our data. This is consistent with the hydrolytic capacity of this organism, with the genome showing a markedly high number of glycoside hydrolases (35). A previous study reported that *Treponema* promotes cellulose degradation by *F. succinogenes* by attaching to solid substrates (18). This may explain the co-occurrence of *F. succinogenes* and *Treponema*.

The relative increase in *F. succinogenes* was very rapid in horses fed the BARLEY diet, with a peak 4 h after the morning meal (corresponding to the arrival of the first digesta), which is not expected for a cellulolytic bacterium (35). However, *F. succinogens* is known to accumulate large amounts of glycogen, representing up to 60–70% of the dry cell mass (7). A possible explanation for this observation is that the accumulation and degradation of glycogen are separated in time. The regeneration of NADP+ from NADPH is generally rate limiting for metabolism under anaerobic conditions. Reducing sugars resulting from starch hydrolysis may be electron and hydrogen acceptors from NADPH through aldehyde reduction (40). However, this needs to be experimentally verified.

The methodological limitations of our study include the relatively shallow sequencing for both the 16S rRNA gene and shotgun metagenome data. Despite shallow sequencing, our results were sufficient to observe the dominant part of the microbiota. Since we performed experiments on large animals, this obviously limited the number of animals investigated. However, the strengths of the study are its longitudinal nature with sampling every hour from the cecum and the crossover design to compensate for the small number of animals examined. Furthermore, the study includes a highly controlled environment and diet.

In conclusion, we herein demonstrated that dynamic microbiota interaction patterns in the horse cecum microbiota switch in a manner that is dependent on diet. We propose that this is due to switches in cross feeding among microbial consortia. An aspect for future research will be to investigate metatranscriptomics and metaproteomics.

## Supplementary tables



## Figures and Tables

**Table 1:** Feed intake (g dry matter) of hay and barley and nutrient intake at 06:00, 14:00, and 22:00 (g meal^−1^) and in total (g d^−1^) when feeding hay only (HAY) or hay supplemented with barley (BARLEY) diets.

Feeding time	HAY	BARLEY	HAY	BARLEY	HAY	BARLEY	HAY	BARLEY
			
	06:00		14:00		22:00		Total	
Dry matter	2400	3610	2400	1780	2400	1780	7200	7170
Ash	130	140	130	100	130	100	390	340
Fat	60	70	60	40	60	40	180	150
Crude protein	220	340	220	170	220	170	660	680
Starch	0	1050	0	0	0	0	0	1050
NDF^1^	1490	1400	1490	1110	1490	1110	4470	3620
ADF^2^	830	690	830	620	830	620	2490	1930

1 NDF: neutral detergent fiber.

2 ADF: acid detergent fiber.
