# Supplementary material for: Diet-Dependent Modular Dynamic Interactions of the Equine Cecal Microbiota
Source: Microbes Environ. 2016 Oct 21;31(4):378–86. doi: 10.1264/jsme2.ME16061 (PMC5158109; doi:10.1264/jsme2.ME16061)
Supplement: Supplementary file 1 [file 31_378_s1.pdf]

Supplementary tables

Supplementary Table S1. Bacterial groups influencing the temporal development.

**Taxonomy**[1)]	**Phylum**	**# of loadings** [1)]	**Dietary differences (p-value)**[2)]
Lachnospiraceae	*Firmicutes*	4	0.004
Anaerostipes	*Firmicutes*	1	<0.001
Anaerovibrio	*Firmicutes*	1	<0.001
Streptococcus	*Firmicutes*	2	<0.001
Fibrobacter s_succinogenes	*Fibrobacteres*	1	<0.001
Treponema	*Spirochaetes*	3	<0.001

1) Number of OTUs with loadings above 0.1 or below -0.1 .

2) p-values calculated by caecum diet based T-tests.

Supplementary Table S2. Relative sequence amount (%) belonging to carbohydrate related functions (mean ± SEM).

**Diet**	BARLEY			HAY		
**Time (h)**	**0**	**4-6[1]**	**23**	**0**	**4-6[1]**	**23**
**Subsystem**						
Central carbohydrate metabolism (2)	19.11±0.28	18.59±0.36	18.78±0.12	18.98±0.14	17.49±0.12	18.73±0.24
Saccharides (2)	16.38±0.26	17.12±0.17	17.05±0.14	16.71±0.15	17.83±0.13	16.77±0.12
One-carbon Metabolism (2)	10.06±0.16	9.83±0.24	10.22±0.12	9.99±0.08	10.38±0.10	10.31±0.11
CO2 fixation (1)	4.89±0.11	5.06±0.13	4.85±0.07	4.85±0.05	4.65±0.07	4.89±0.11
Fermentation (1)	4.80±0.09	5.40±0.33	4.96±0.04	4.66±0.04	4.46±0.10	4.95±0.08
Sugar alcohols (1)	3.04±0.11	2.94±0.06	2.90±0.07	2.95±0.04	2.67±0.05	2.92±0.04
Aminosugars (1)	2.57±0.04	2.21±0.18	2.38±0.07	2.55±0.06	2.31±0.03	2.37±0.03
Glycoside hydrolases (1)	0.57±0.04	0.73±0.13	0.48±0.02	0.52±0.02	0.68±0.02	0.54±0.01

[1] Corresponding to the time point for pH minimum

Supplementary Table S3 T-test calculated p-values.

	**Diet comparison at different time points**			**Time point comparison in hay diet**			**Time point comparison in hay+barely diet**			**Total diet comparison**
**Metabolic feature** / **Time points (h)**	**0**	**4-6**	**233**	**0-(4-6)**	**(4-6)-23**	**0-13**	**0-(4-6)**	**(4-6)-23**	**0-13**	
**Aminosugars**	0.746	0.594	0.940	0.083	0.410	0.043	0.004	0.181	0.023	0.762
**CO2 fixation**	0.756	0.017	0.766	0.309	0.181	0.793	0.047	0.092	0.729	0.091
**Central carbohydrate metabolism**	0.696	0.020	0.854	0.277	0.628	0.312	<0.0005	0.001	0.381	0.072
**Fermentation**	0.210	0.025	0.877	0.116	0.225	0.140	0.105	0.002	0.008	0.011
**Glycoside hydrolases**	0.281	0.741	0.036	0.280	0.100	0.073	<0.0005	<0.0005	0.288	0.853
**Monosaccharides**	0.295	0.006	0.153	0.032	0.746	0.043	<0.0005	<0.0005	0.721	0.176
**One-carbon Metabolism**	0.701	0.064	0.569	0.437	0.178	0.444	0.011	0.664	0.036	0.131
**Sugar alcohols**	0.467	0.004	0.807	0.453	0.659	0.309	0.001	0.001	0.621	0.063

-- Between time points.

Supplementary figures

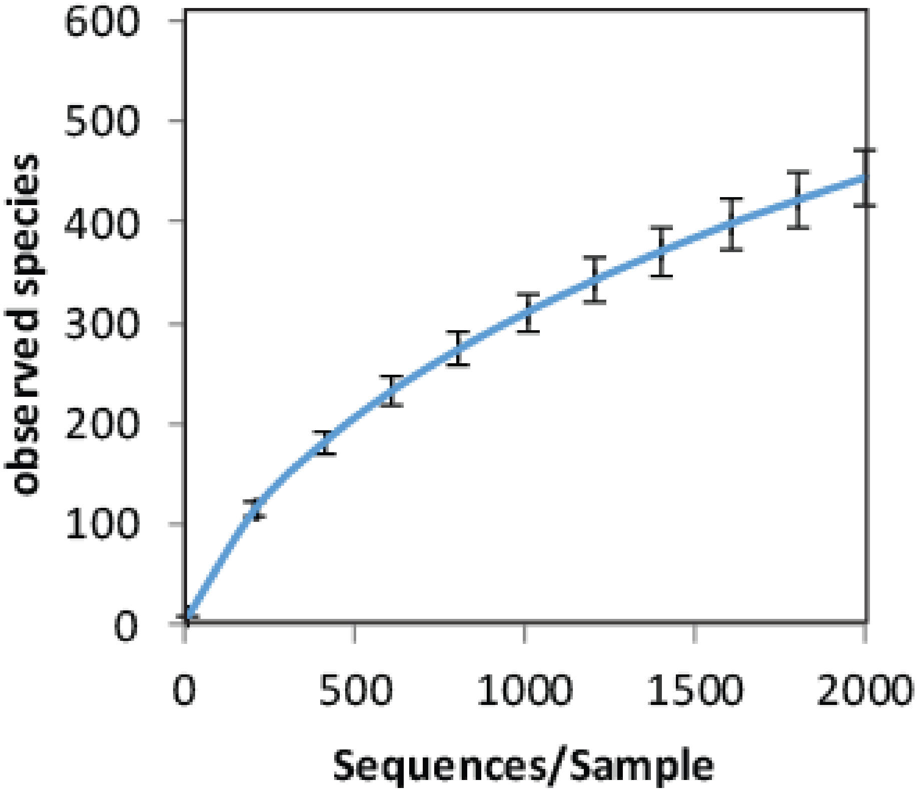

Supplementary Figure S1. Rarefaction curve for observedOTU's (mean + SEM)

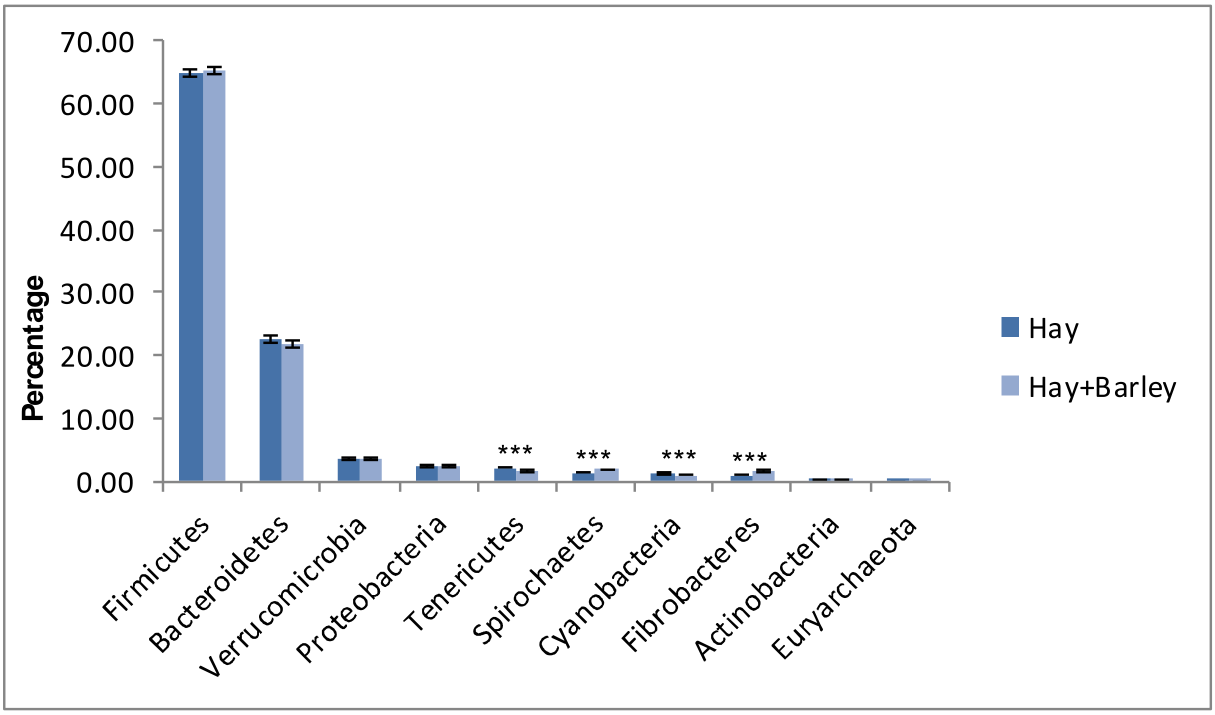

Supplementary Figure S2. Phyla distribution according to diet (mean + SEM). Significance is indicated by ***p<0.001).

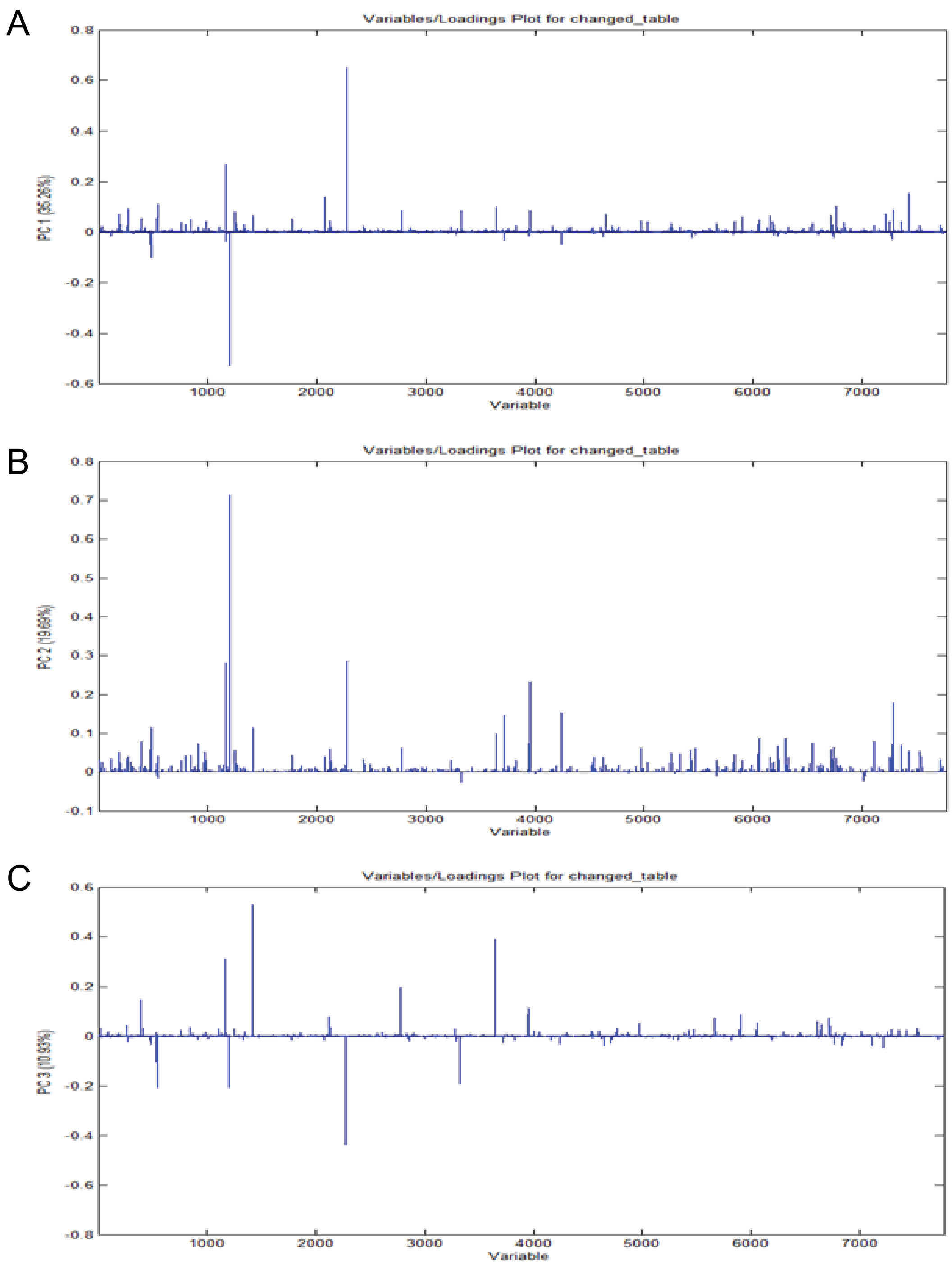

Supplementary Figure S3. Loadings plot according to PC1 (A), PC2 (B) and PC3 (C) for cecum samples. The variables correspond to the respective OTU's.

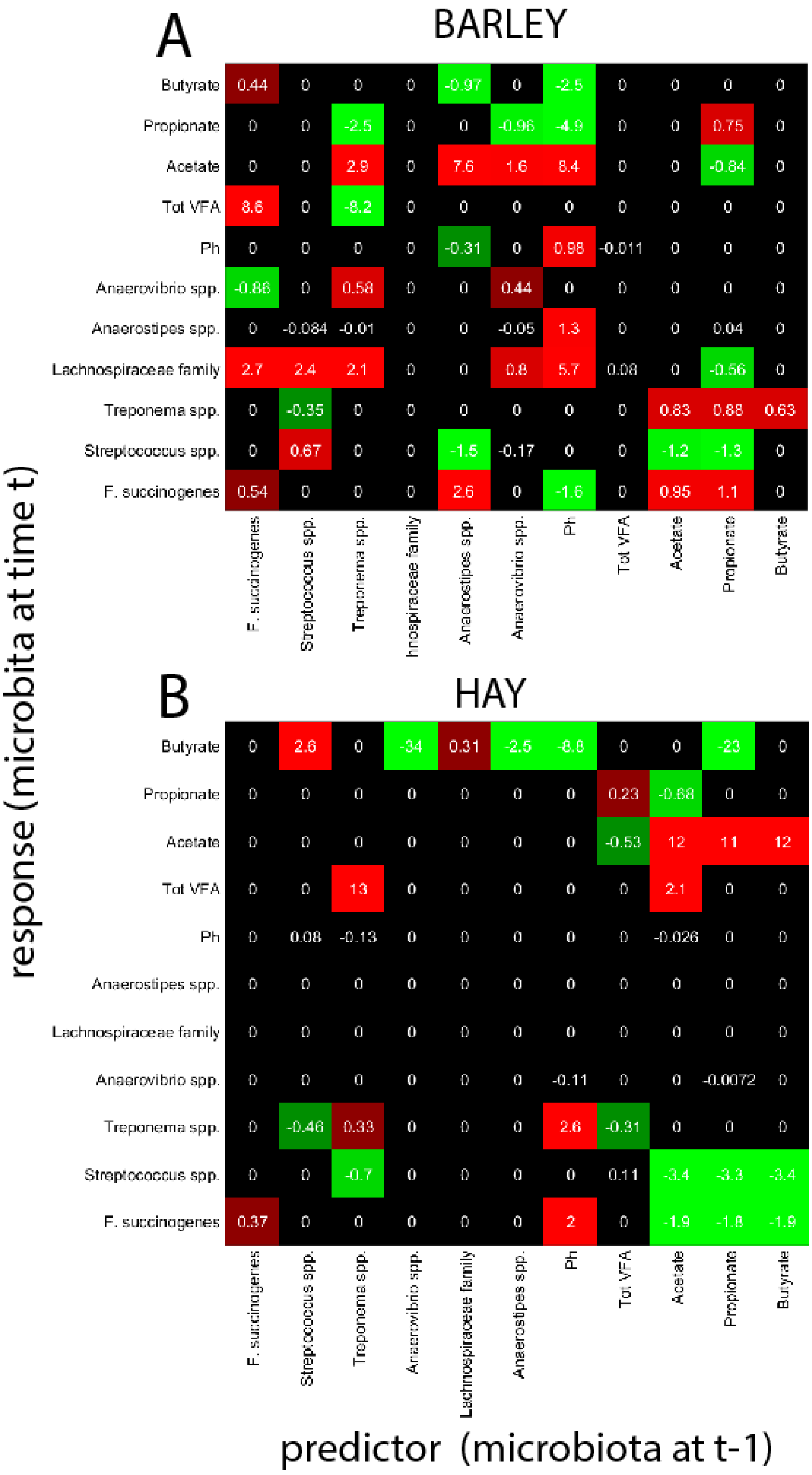

Supplementary Figure S4. Dynamic interactions in the horse caecum when feeding hay supplemented with barley (A) or hay only (B) diets. The dynamic interactions were modelled independently for each of the response variables at time t, where all microbiota components at time t-1 were used as predictors for each of the responses at time 1. The numeric values represent the regression coefficients for each of the significant predictor variables. The color coding indicates the direction, where red represents positive and green negative values.

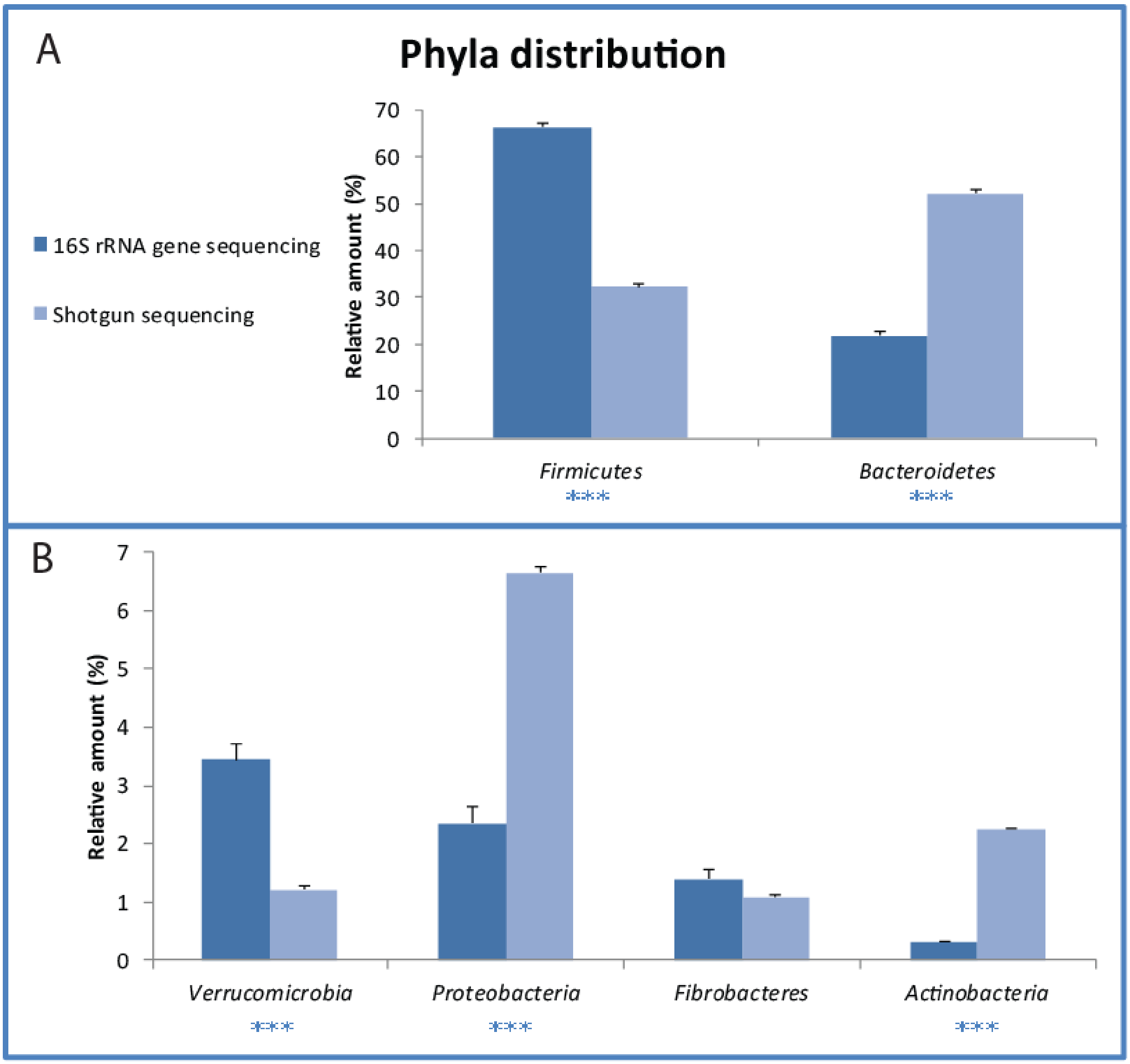

Supplementary Figure S5. Phyla distribution comparison between 16S rRNA gene sequencing and shotgun sequencing in respective samples (mean + SEM). A) Most dominating phyla *Firmicutes* and *Bacteroidetes*. **B)** Remaining common phyla representing more than 1% of the total bacterial content with shotgun sequencing and respective phyla in 16S rRNA gene sequencing. ***Significant difference between the two methods at the specific phyla ($p<0.001$).

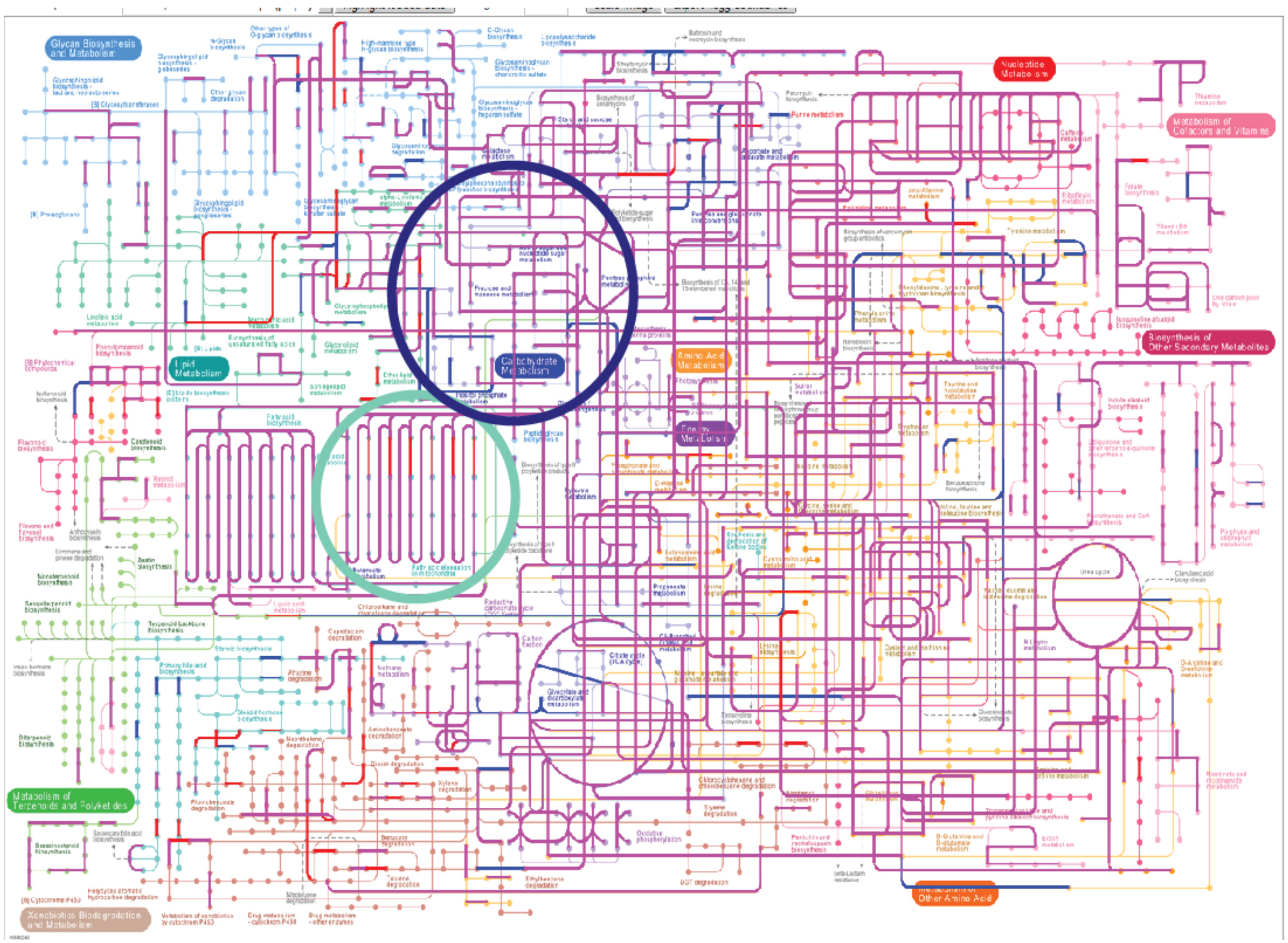

Supplementary Figure S6. KEGG metabolic map for differences between metabolic pathways between. Pathways that were overrepresented for the BARLEY diet are marked with blue, the pathways overrepresented for the HAY diet are marked with red, while the overlapping pathways are purple. The map was generated using the KEGG mapper plugin in MG-rast. The blue circle marks important carbohydrate pathways, while the turquoise map the fatty acid chain elongation pathway.

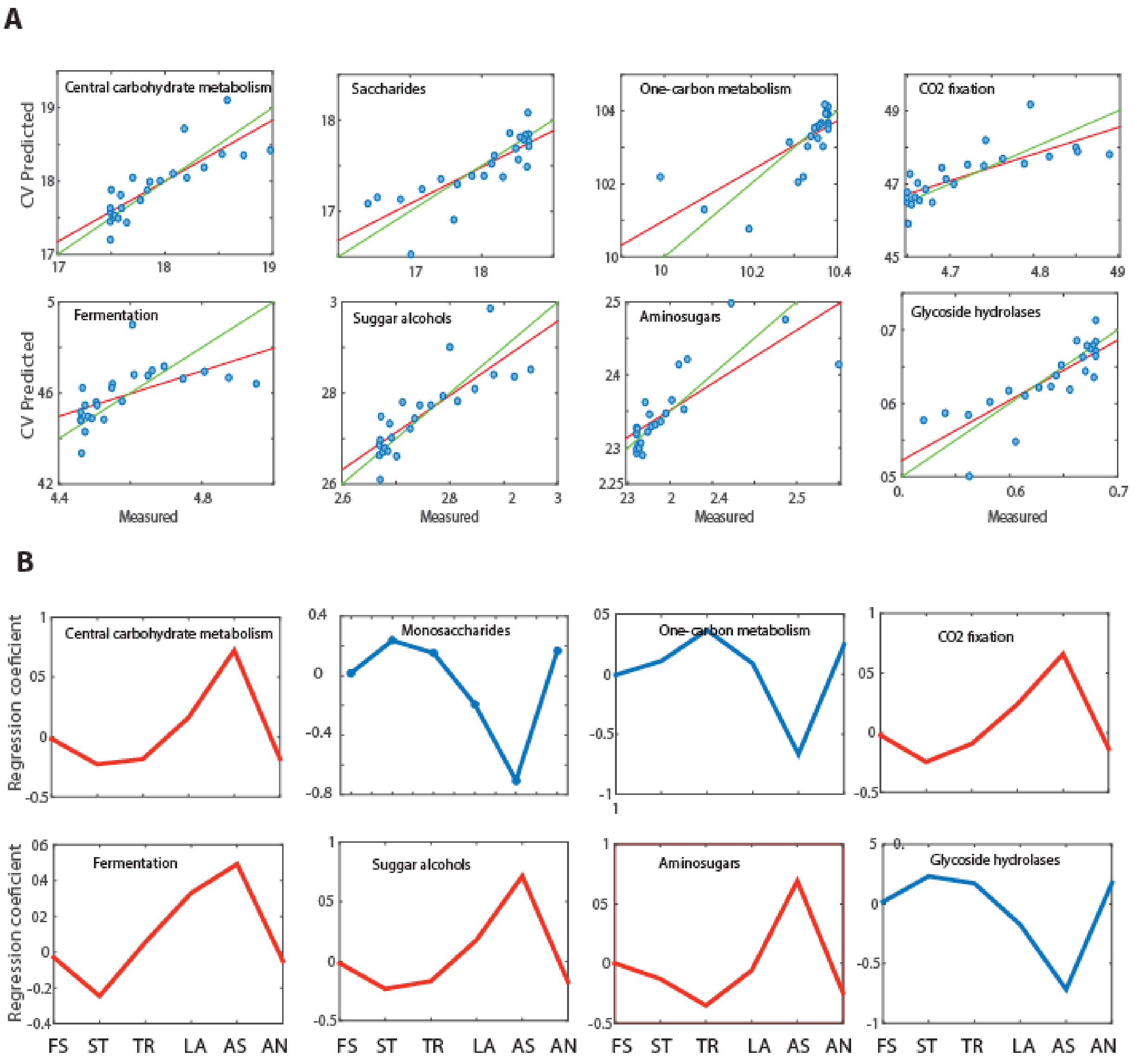

Supplementary Figure S7. BARLEY diet prediction of carbohydrate subsystems based on the microbiota composition (A), with the corresponding regression vector (B). The predictions done by PLS based on average interpolated levels of carbohydrate subsystems and the bacterial loads across the 4 horses over the 24 hour span. Venetian blinds cross-validation was used. (A)The red lines represent the cross-validated regression, while the green represent the midpoints. (B) The two patterns detected as outlined in Fig. 5 are marked with red and blue lines, respectively. The following abbreviations are used; LA: Lachnospiraceae, AS: Anaerostipes, AN: Anaerovibrio. ST: Streptococcus, FS: Fibrobacter succinogenes and TR: Treponema.

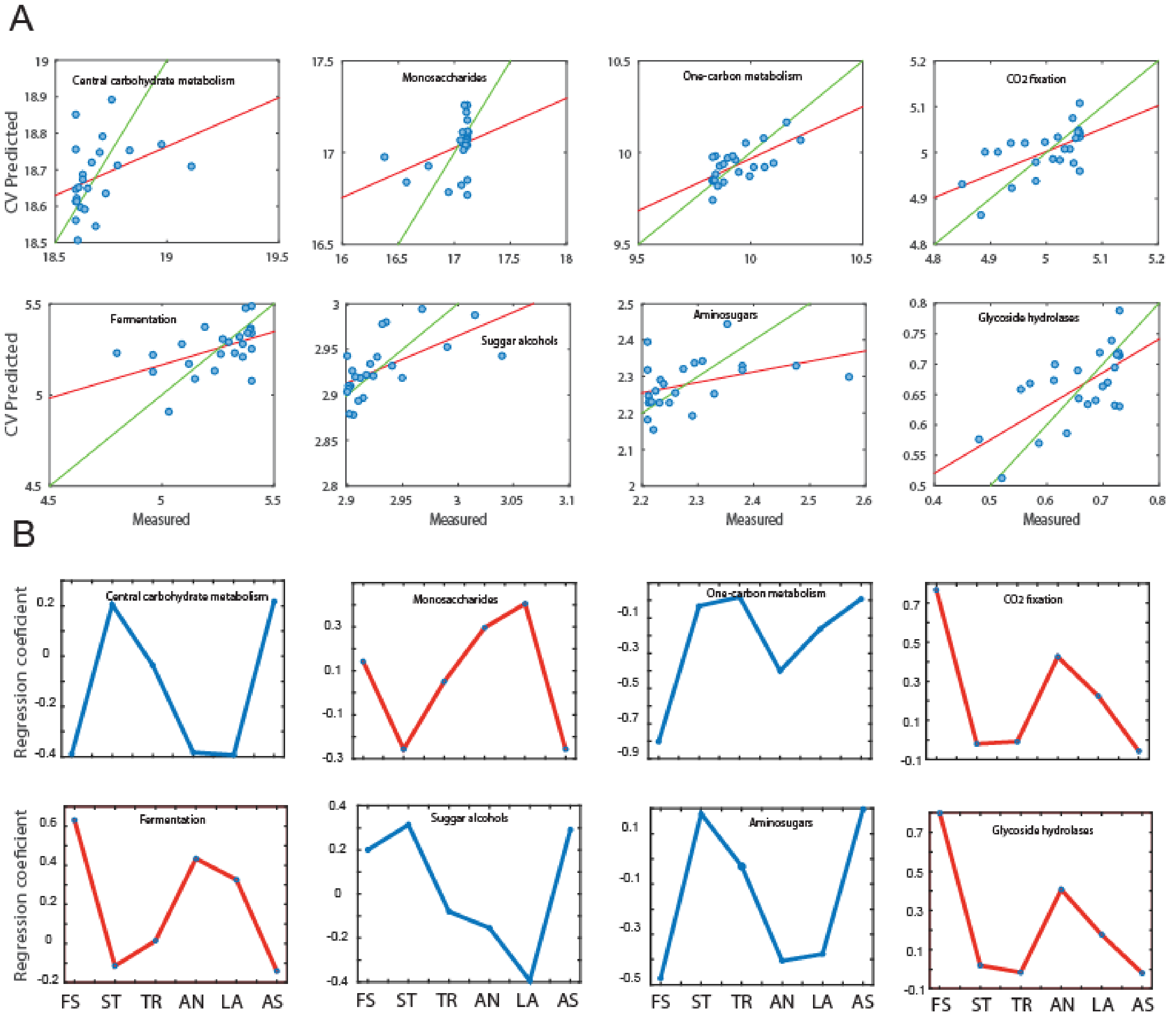

Supplementary Figure S8. HAY diet prediction of carbohydrate subsystems based on the microbiota composition (A), with the corresponding regression vector (B). The predictions done by PLS based on average interpolated levels of carbohydrate subsystems and the bacterial loads across the 4 horses over the 24 hour span. Venetian blinds cross-validation was used. (A) The red lines represent the cross-validated regression, while the green represent the midpoints. (B) The two patterns detected as outlined in Fig.5 are marked with red and blue lines, respectively. The following abbreviations are used; LA: Lachnospiraceae, AS: Anaerostipes, AN: Anaerovibrio. ST: Streptococcus, FS: Fibrobacter succinogenes and TR: Treponema.
